# 820. Feasibility of Ultra-portable Negative Pressure Wound Therapy in Acute Pediatric Burns: Pilot Randomized Controlled Trial

**DOI:** 10.1093/jbcr/irag033.259

**Published:** 2026-04-06

**Authors:** Bronwyn R Griffin, Emma R Lumsden, Roy M Kimble, Robert Ware

**Affiliations:** Griffith University, Brisbane, QLD; Queensland Children's Hospital, Brisbane, QLD; Queensland Children's Hospital, Brisbane, QLD; Griffith University, Brisbane, QLD

## Abstract

**Introduction:**

Negative Pressure Wound Therapy (NPWT) has been shown to decrease scar formation in acute pediatric burns, but the large size of conventional NPWT devices can limit their acceptability in children. A smaller, ultra-portable NPWT device may reduce this treatment burden while preserving the benefits of NPWT. This study aimed to evaluate the feasibility, safety, and clinical application of ultra-portable NPWT in the management of minor pediatric burns.

**Methods:**

We conducted a single-center, three-arm, parallel randomized controlled pilot trial at a quaternary pediatric burns unit. Children <16 years with minor burns suitable for the ultra-portable NPWT dressing dimensions were eligible. Participants were randomized to one of three groups: (1) silicone + silver nanocrystalline; (2) silicone + silver nanocrystalline + ultra-portable NPWT; or (3) silicone + silver nanocrystalline (thin) + ultra-portable NPWT. The primary outcome was feasibility, defined by recruitment, treatment fidelity, data completeness, and study completion. Secondary outcomes included the feasibility of the ultra-portable NPWT application, patient/clinician perceptions, complication rates, and the impact on clinical workload.

**Results:**

Thirty-one participants were randomized (screening rate: 5.6%). All feasibility benchmarks were achieved except recruitment (76.5% vs the prespecified 80%). Dressing feasibility did not differ between groups. Complications were significantly higher in the silicone + regular silver nanocrystalline + ultra-portable NPWT group compared with silicone + silver nanocrystalline alone (*p*<.001). In contrast, ultra-portable NPWT combined with silver nanocrystalline (thin) did not increase complications (*p*=.1). Both ultra-portable NPWT groups increased clinician workload compared with standard dressings (*p*=.01 for silver nanocrystalline, *p*=.04 for silver nanocrystalline (thin)).

**Conclusions:**

Ultra-portable NPWT can be feasibly trialed and clinically applied in select pediatric burn patients, though its use is limited by patient eligibility and clinician workload. Future studies should prioritize combinations with silver nanocrystalline (thin) rather than regular silver nanocrystalline, to minimize complications.

**Applicability of Research to Practice:**

This is the first randomized study to test the feasibility of ultra-portable NPWT in pediatric burns, addressing the need for portable, child-friendly NPWT options. Findings guide clinical application and design of larger trials, with the potential to broaden NPWT accessibility and reduce long-term burn scarring in children.

**Funding for the study:**

N/A.

